# Comprehensive evaluation of candidate reference genes for quantitative real-time PCR-based analysis in Caucasian clover

**DOI:** 10.1038/s41598-021-82633-2

**Published:** 2021-02-08

**Authors:** Xiujie Yin, Taotao He, Kun Yi, Yihang Zhao, Yao Hu, Jiaxue Liu, Xiaomeng Zhang, Lingdong Meng, Lina Wang, Haoyue Liu, Yonggang Li, Guowen Cui

**Affiliations:** College of Animal Science and Technology, Northeast Agricultural University, No. 600 Changjiang Street, Xiangfang District, Harbin City, Heilongjiang China

**Keywords:** Genetics, Physiology, Plant sciences

## Abstract

The forage species Caucasian clover (*Trifolium ambiguum* M. Bieb.), a groundcover plant, is resistant to both cold and drought. However, reference genes for qRT-PCR-based analysis of Caucasian clover are lacking. In this study, 12 reference genes were selected on the basis of transcriptomic data. These genes were used to determine the most stably expressed genes in various organs of Caucasian clover under cold, salt and drought stress for qRT-PCR-based analysis. Reference gene stability was analyzed by geNorm, NormFinder, BestKeeper, the ∆Ct method and RefFinder. Under salt stress, *RCD1* and *PPIL3* were the most stable reference genes in the leaves, and *NLI1* and *RCD1* were the most stable references genes in the roots. Under low-temperature stress, *APA* and *EFTu-GTP* were the most stable reference genes in the leaves, and the *RCD1* and *NLI2* genes were highly stable in the roots. Under 10% PEG-6000 stress, *NLI1* and *NLI2* were highly stable in the leaves, and *RCD1* and *PPIL3* were the most stable in the roots. Overall, *RCD1* and *NLI2* were the most stable reference genes in organs under normal conditions and across all samples. The most and least stable reference genes were validated by assessing their appropriateness for normalization via *WRKY* genes.

## Introduction

Caucasian clover (*Trifolium ambiguum* M. Bieb.), also known as Kura clover, is a high-quality rhizomatous perennial legume forage and groundcover species that originated in Russia and is distributed in areas with a cool and humid climate^1^. The dense rhizome system of Caucasian clover can store large amounts of metabolic energy that enables the perennial persistence and rapid reestablishment of this species^2^, and a unique characteristic of this species is the formation of daughter plants from the rhizomes that develop from the taproot. This characteristic confers an advantage to Caucasian clover under drought conditions^3^. To overcome abiotic stress (salt, cold, heat, and drought) and biotic stress (pests and pathogens), various mechanisms have evolved, but the molecular bases of these mechanisms are complex. A draft transcriptome of Caucasian clover has been assembled, mapped and functionally annotated and is available via public databases (accession number SRP159097 in the NCBI database). Nonetheless, few studies have focused on Caucasian clover gene expression. More research on the molecular mechanisms in Caucasian clover is needed.

The discovery of key genes involved in certain biological processes is the most important step in molecular research^4^. Several techniques are available to investigate gene expression, including semiquantitative reverse transcription polymerase chain reaction, northern blotting, in situ hybridization, and quantitative real-time PCR (qRT-PCR)^5^. A substantial amount of molecular research on plants has aimed to elucidate various processes through related functional genes. qRT-PCR is the most common technique used for validation of data from transcriptomic studies because of its reproducibility, rapidity, accuracy and sensitivity^6^.

Reference genes, also called housekeeping genes, are expressed stably in different tissues during different physiological states of organisms and in response to different environmental stimuli^7^. Ideal reference genes that are stably expressed during different biological and physiological states can be effectively used for normalization^8^. Several reference genes, including actin (*ACT*), elongation factor 1 alpha subunit (*EF1α*), glyceraldehyde-3-phosphase (*GAPDH*) and tubulin alpha (*TUA*), have been utilized for reliable qRT-PCR-based studies in various species^9–12^. Many reports have indicated that ideal stable reference genes do not exist and that stable reference genes differ among plant species, growth stages, growth conditions and treatments^13^. In addition, numerous studies have reported reference genes in various species, including *Miscanthus lutarioriparia*^14^, ladybird beetle^15^, pitaya^16^ and *Hemarthria compressa*^17^. Moreover, *UBQ* was found to be the most stable of seven candidate genes across all organs (leaves and stolons) and treatments (water-limited and well-watered conditions) in white clover (*Trifolium repens* L.)^18^, and *Tr-β-ACTIN* and *Tr-GAPDH* are two reference genes whose transcript abundance remains stable under biotic stress conditions in white clover^19^*.*

There are no reports in the literature on the evaluation of candidate reference genes for qRT-PCR in Caucasian clover. Thus, in this study, we selected 12 candidate reference genes: those encoding cell differentiation protein *RCD1* homolog (*RCD1*)^20^, CYP, protein phosphatase type 2A complex (*PP2A*)^21^, *NLI* interacting factor-like phosphatase (*NLI1*), NLI interacting factor (*NLI2*), elongation factor *Tu GTP*-binding domain (*EFTu-GTP*), cationic amino acid transporter 9 (*APA*), *RBD*, protein tyrosine phosphatase activity (*PTPMT1*), tobamovirus multiplication-like protein (*TMP*), microtubule-associated protein *CRIPT* (*MAP*)^21^ and a *bZIP* transcription factor (*BZIP*). To investigate the most appropriate genes for normalization in this species and confirm the suitability of these genes, the relative expression levels of the target genes of *WRKY* were analyzed via qRT-PCR, with the most and least stable reference genes used. The results of this study will advance research on Caucasian clover.

## Material and methods

### Plant materials, growth conditions and abiotic stress treatments

Caucasian clover seedlings were used in this study. Original sources of the plant materials were obtained from the Inner Mongolia Grass Variety Engineering Technology Research Center of Inner Mongolia Agricultural University. Staff at the center formally identified the samples, provided details of specimen deposition and provided seeds (International Plant Name Index (IPNI) Life Sciences Identifier (LSID) urn:ipni.org:names:522843-1). After they were sterilized with 75% ethanol for 30 s, followed by NaClO_4_ for 10 min, the seeds were sown in vermiculite in plastic pots (10 cm diameter, 9 cm depth) in a greenhouse at Northeast Agricultural University on 9 September 2018. The greenhouse had a day/night average temperature of 24/18 °C, a photoperiod of 16/8 h (light/dark) and a relative humidity of 70–80%, and the vermiculite was kept moist with 1/2-strength Hoagland nutrient solution. The plants were treated when they were 5 weeks old. Samples from different organs (roots, stems and leaves) were collected from the plants for 5 weeks. The experimental design included three biological replicates.

Five treatments were applied in the present study: (1) a low-temperature treatment involving subjecting the plants to 4 °C for different durations (0, 2, 6, 12, and 24 h) under irrigation with Hoagland solution, after which leaf and root tissues were collected; (2) a salt treatment involving the application of 200 mL of NaCl at different concentrations (0, 12.5, 25, 50 and 100 mmol/L) to simulate high-salt conditions for 4 h, after which leaf and root tissues were collected; (3) a drought stress treatment involving subjecting plants to 10% polyethylene glycol (PEG)-6000 solution (w/v; Sangon, China) for different durations (0, 2, 6, 12, and 24 h), after which leaf and root tissues were collected; (4) a control in which stems, leaves and roots were collected under normal conditions; and (5) a treatment in which all samples from the four treatments were considered together for all samples. All the samples were collected in triplicate, frozen in liquid nitrogen, and stored at − 80 °C.

### Total RNA isolation and first-strand cDNA synthesis

Total RNA was extracted from each sample by TRIzol reagent (Invitrogen, Carlsbad, CA, USA) according to the manufacturer’s instructions. Genomic DNA (gDNA) was removed by RNase-free DNase I digestion during the isolation procedure. The quantity and purity of RNA were determined via 1% agarose gel electrophoresis. RNA whose A260/280 ratio ranged from 1.8 to 2.2 and whose A260/230 ratio was greater than 2.0 was used for further synthesis. All the RNA samples were stored at − 80 °C. For qPCR, first-strand cDNA was synthesized from 1 μg of total RNA via HiScript II Q RT SuperMix for qPCR (+ gDNA wiper) according to the manufacturer’s protocol^7^. Before the next step, the cDNA samples were stored at − 20 °C.

### Selection of candidate reference genes, mining of target transcripts, designing of primers and verification of selected gene amplicons

The RNA transcriptome sequence of Caucasian clover is available from a public database (accession number SRP159097 in the NCBI database). Twelve candidate reference genes among 27,004 genes (with fragments per kilobase of transcript per million mapped reads (FPKM) values ≥ 5 for at least three replicates per treatment and differences from each other not surpassing 5)^22^ identified via RNA sequencing analysis were selected from the RNA transcriptome sequence of Caucasian clover. In addition, data mining for target transcripts within the transcriptome was also performed.

**Figure 1:**
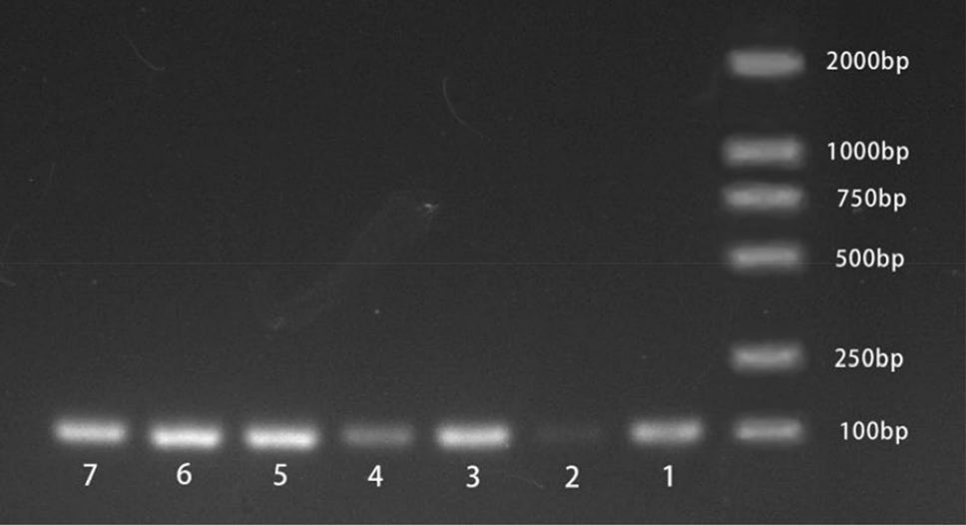
Specificity of Caucasian clover reference gene primer pairs for RT-qPCR amplification. Agarose gel (1.0%) electrophoresis displaying single PCR products with expected sizes for the 12 reference genes (right side, 50 bp DNA ladder). Figures 1–7 represent the *RCD1*, *PPIL3*, *PP2A*, *NLI1*, *NLI2*, *EFTu-GTP* and *RBD* reference genes, respectively, and the other reference genes identified via agarose gel electrophoresis are displayed in Fig. S1 of the additional files.

The primer pairs of candidate reference genes and target transcripts were designed via the NCBI website (https://www.ncbi.nlm.nih.gov/) and Oligo 6 software, with the following parameters: a primer length of 20–22 bp (optimal length of 100 bp), an amplicon length of 80–150 bp, an annealing temperature (Tm) within the range of 57–63 °C (optimal temperature of 60 °C), and a temperature difference of each primer of less than 1 °C. All the primers used were synthesized by a commercial supplier (RiboBio, Harbin, China). All the information about the primer design in this study is listed in Table 1. Primer specificity was determined via 1.0% agarose gel electrophoresis (Fig. 1 and Fig. S1).

**Table 1:** Caucasian clover candidate reference genes, primer sequences, amplicon sizes, melting temperatures (T_ms_), amplification efficiencies (E_s_), and regression coefficients (R^2^_s_).

Gene ID	Gene name	Description	Primer sequence (5′–3′): forward/reverse	Amplicon size (bp)	Melting temperature (T_m_)	E (%), amplification efficiency	R^2^, correlation coefficient
c245114.graph_c1	RCD1	Cell differentiation protein RCD1 homolog	CTACAGCGAAAAAGCGCTCA/ATAATTCCCCTGTGCCTGCG	142	82.2	1.0186	0.9725
c250723.graph_c0	PPIL3	CYP	ATGCGAAATCTTCTGCGACG/ACCAGTTGGGTCTCCACCTT	136	79.9	0.9295	0.9676
c251578.graph_c0	PP2A	Protein phosphatase type 2A complex	TCTGCTCCCCGTCTTACAAC/AGACGACAAGTTCCAACCCC	80	82.7	0.9295	0.9913
c253856.graph_c0	NLI 1	NLI interacting factor-like phosphatase	TACCGGAGTGAACGTTGTCC/ACGAAGCGTGGAGGTTGATT	124	79.4	1.0148	0.9830
c256191.graph_c0	NLI 2	NLI interacting factor	TAGACGGGCTTTGTACCCCA/CCCAGAAGGTGACAGTACGC	113	80.8	0.9500	0.9870
c258522.graph_c0	EFTu-GTP	Elongation factor Tu GTP-binding domain	GTTTCCATGCGTGTGTCGTC/CCACCCTTCCTTCATCGGAC	104	82.1	0.9362	0.9793
c259373.graph_c0	RBD	RBD domain	CCCACGTCTGGATGAGCTAC/TCGCTTTGCTGGAGACTGTT	148	79.6	0.9128	0.9801
c262622.graph_c0	APA	Cationic amino acid transporter 9	GCCACACCCCTCTTCATTCT/TAGCCAGTCAGCGTACCAAC	115	79.5	0.9746	0.9957
c263978.graph_c0	PTPMT1	Protein tyrosine phosphatase activity	AAGTCAATTCGGCCAAGGGT/CCAACAGCCCTCCTCACAAT	92	80.8	0.9853	0.9959
c265061.graph_c0	TMP	Tobamovirus multiplication-like protein	GGCTGTGACAAATCCGACCT/TAGCTGCTGCACTAGGCTTC	119	79.0	1.0338	0.9994
c268107.graph_c0	MAP	Microtubule-associated protein CRIPT	AACTTGCTTGCCACACATCG/TGGACCCCTTACGGAACTACT	120	79.8	1.0148	0.9778
c269312.graph_c1	bZIP	bZIP transcription factor	AATTCCGACGATCACCCACC/TTCCACCTCGTAAAGGGCAC	102	80.7	0.9328	0.9943
c261427.graph_c0	WRKY	WRKY family transcription factor	CTTCGTTACCGGAGTCCCCT/TGGATGGCTCCGTCAAAGTC	70	80.5	0.9675	0.9978

### qRT-PCR and gene-specific PCR amplification efficiency

qRT-PCR was performed in 96-well plates on a Quantagene q225 Real-Time PCR System (Novogene). The total volume of the reaction solution was 10 μL, which consisted of 0.5 μL of cDNA, 0.2 μL of forward primer, 0.2 μL of reverse primer, 4.1 μL of ddH_2_O and 5 μL of 2× ChamQ Universal SYBR qPCR Master Mix. The PCR procedure was as follows: the thermal profile of the reaction included initial denaturation at 95 °C for 2 min, followed by 40 cycles at 95 °C for 15 s and 57 °C for 30 s and a final step of 72 °C for 15 s. A melting curve was produced after 40 cycles of amplification by heating at 65–95 °C to confirm the specificity of the PCR products. Slopes in the range of − 3.58 to − 3.10 were considered acceptable for the PCR assay.

The amplification efficiency E = 10^(−1/slope of the standard curve)^ and R2 value for all primer pairs were determined from a 6-point standard curve produced by tenfold serial dilutions of cDNA, in triplicate^23^.

### Ranking the stability of candidate reference genes

geNorm^23^, NormFinder^24^, BestKeeper^25^, the ΔCt method and RefFinder^26^ (https://www.heartcure.com.au/reffinder/?type=reference) were used to analyze the stability of the candidate reference genes under different conditions. All procedures were performed in accordance with the program instructions.

The geNorm algorithm, described by Vandesompele et al.^27^, calculates the gene expression stability, M, for a reference gene as the average pairwise variation, V, for that gene with all other tested reference genes. Stepwise exclusion of the gene with the greatest M value allows ranking of the tested genes according to their expression stability. Stable reference genes with M values lower than 1.5 can be used with geNorm to determine the optimal number of reference genes for normalization by the pairwise variation V_n/n+1_. Variations with values below the threshold of 0.15 are considered ideal pairwise variations^28,29^.

NormFinder software was used to identify the stable reference genes, and the principle underlying the calculations was similar to that used by geNorm. First, we obtained the expression stability, M, and then selected the most stable reference gene according to M. The standard was the most stable reference gene with the smallest M value. However, this program selects only the most suitable reference gene.

The BestKeeper algorithm compares the expression levels of only ten reference genes and ten target genes in 100 samples. The correlation coefficient (r), standard deviation (SD) and coefficient of variation (CV) between each gene were obtained. The magnitudes of the respective values were then compared, after which the reference gene with the best stability was ultimately determined.

With the ΔCt method, ranks were determined according to pairwise comparisons of gene sets. The reference gene with the lowest SD had the most stable expression. RefFinder was then used to combine all four statistical methods (geNorm, NormFinder, BestKeeper and the ΔCt method) to calculate the comprehensive ranks.

### Validation of reference gene stability

In the present study, the *WRKY* gene was verified to be involved in various processes, including responses to biotic and abiotic stresses. The relative expression data were calculated according to the 2^−ΔΔCt^ method^30^ and presented as relative expression levels. The sequence of *WRKY* was obtained from the RNA sequence of Caucasian clover deposited in the NCBI database. In this study, we used *WRKY* as a target gene to validate the stability of the reference genes. The relative expression levels of *WRKY* in the roots of plants under low-temperature treatment and in the roots of plants under salt stress were determined and normalized by the use of the most and least stable reference genes.

## Results

### Primer verification and expression levels of candidate reference genes

**Figure 2:**
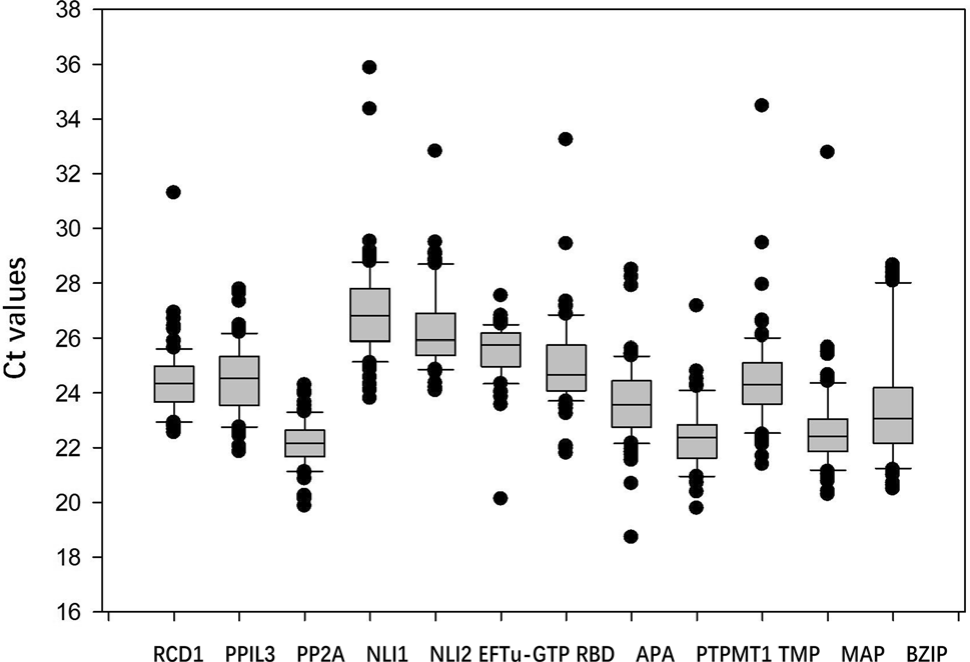
qRT-PCR Ct values of 12 candidate reference genes across all samples. The box graph indicates the 25th and 75th percentiles. The line across the box depicts the median. The lower and upper dashes represent the minimum and maximum values, respectively.

We determined the cycle threshold (Ct) values for 12 candidate reference genes (three biological replicates and three technical replicates). The Ct values of all the candidate reference genes are shown in Supplementary Table S1 and Fig. 2, and the expression levels of the candidate reference genes were measured. The Ct values ranged from 22.15 (*PP2A*) to 27.04 (*NLI1*). The variable Ct values of all the candidate reference genes among the different treatment conditions demonstrated that the expression levels varied on the basis of the conditions and experimental treatments. The expression of *PP2A* (Ct = 22.15), followed by that of *PPIL3* (Ct = 24.55), *RCD1* (Ct = 24.58) and *NIL2* (Ct = 26.35), was little affected by the treatments, whereas the expression of *NLI1* (Ct = 27.04) was strongly affected by the treatments. These results implied that the expression of all the reference genes was inconsistent across all the treatments and experimental conditions. Thus, it was necessary to select the most stable reference genes for normalizing gene expression in Caucasian clover under different conditions (Fig. 2).

### Expression stability of candidate reference genes

To further evaluate the stability of the candidate reference genes, we used five methods (geNorm, NormFinder, BestKeeper, the ∆Ct method and RefFinder) to determine the individual expression stability of the genes.

### geNorm analysis

**Figure 3:**
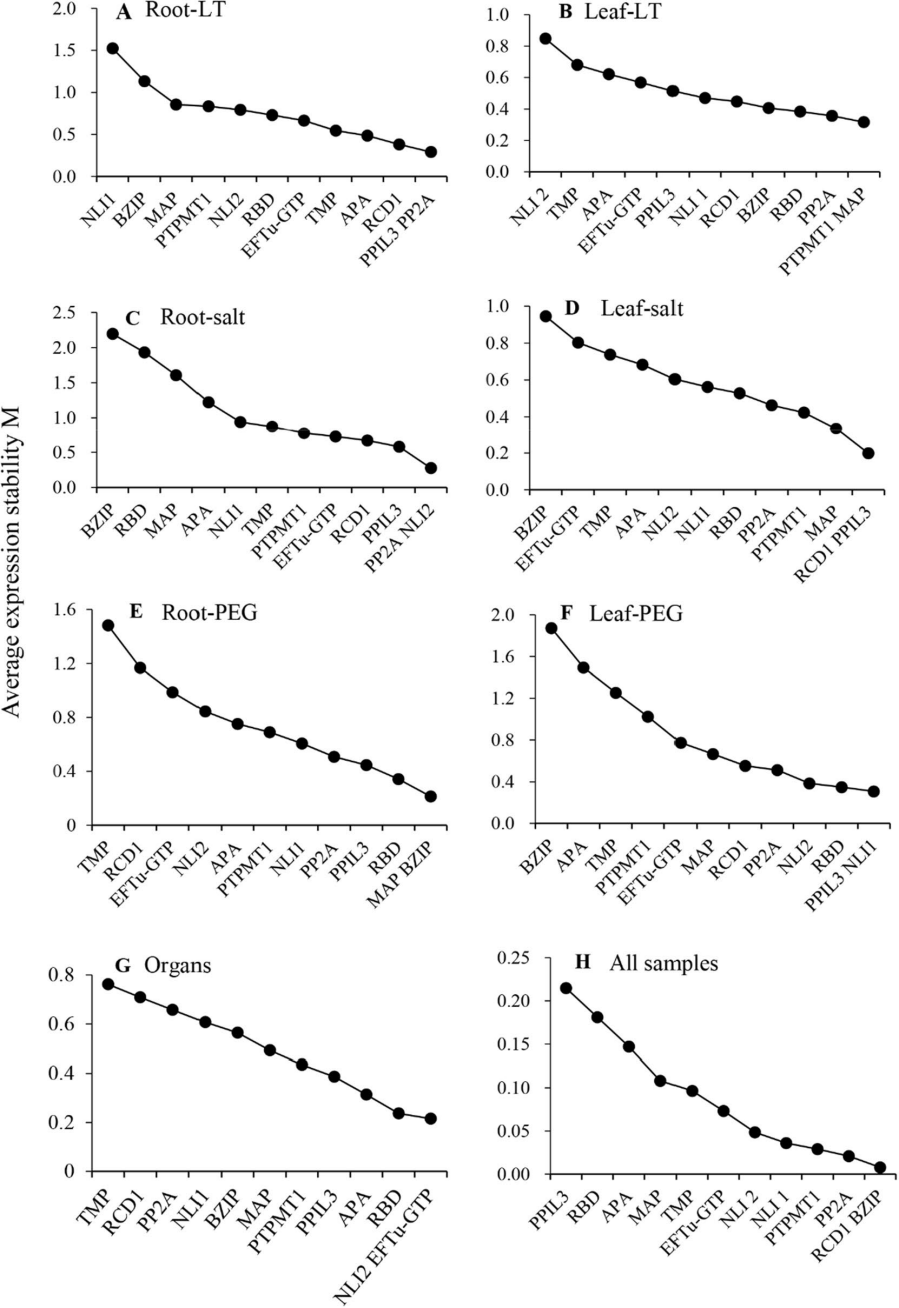
Average M values of the 12 candidate reference genes according to geNorm. The expression stability was evaluated in samples from organs, in all samples, and in the roots and leaves of Caucasian clover subjected to low-temperature, salt and 10% PEG treatments. The least stable reference genes, which have relatively high M values, are shown on the left, and the most stable reference genes, which have relatively low M values, are shown on the right.

In the geNorm analysis, the M values were determined for root and leaf samples of plants subjected to three different treatments (Fig. 3). The rank of the candidate reference genes differed on the basis of the different conditions. In the roots of plants under low temperature (Fig. 3A), *PP2A* and *PPIL3* had the lowest M values, which indicated that they were most stable under the low-temperature treatment. *NLI1* had the greatest M value (1.51), and its expression was the most unstable. In the leaves of plants under low temperature (Fig. 3B), all of the M values were below the threshold of 1.5, and the expression of *PTPMT1* and *MAP* was the most stable. Overall, M is suggested to be the criterion for appropriate reference gene selection (Fig. 3). In the roots of plants under salt treatment, the M values of *BZIP*, *RBD* and *MAP* were > 1.5. In the leaves of plants under 10% PEG-6000 conditions, the M value of *BZIP* was > 1.5; all the other genes were perhaps more suitable reference genes for target gene normalization.

**Figure 4:**
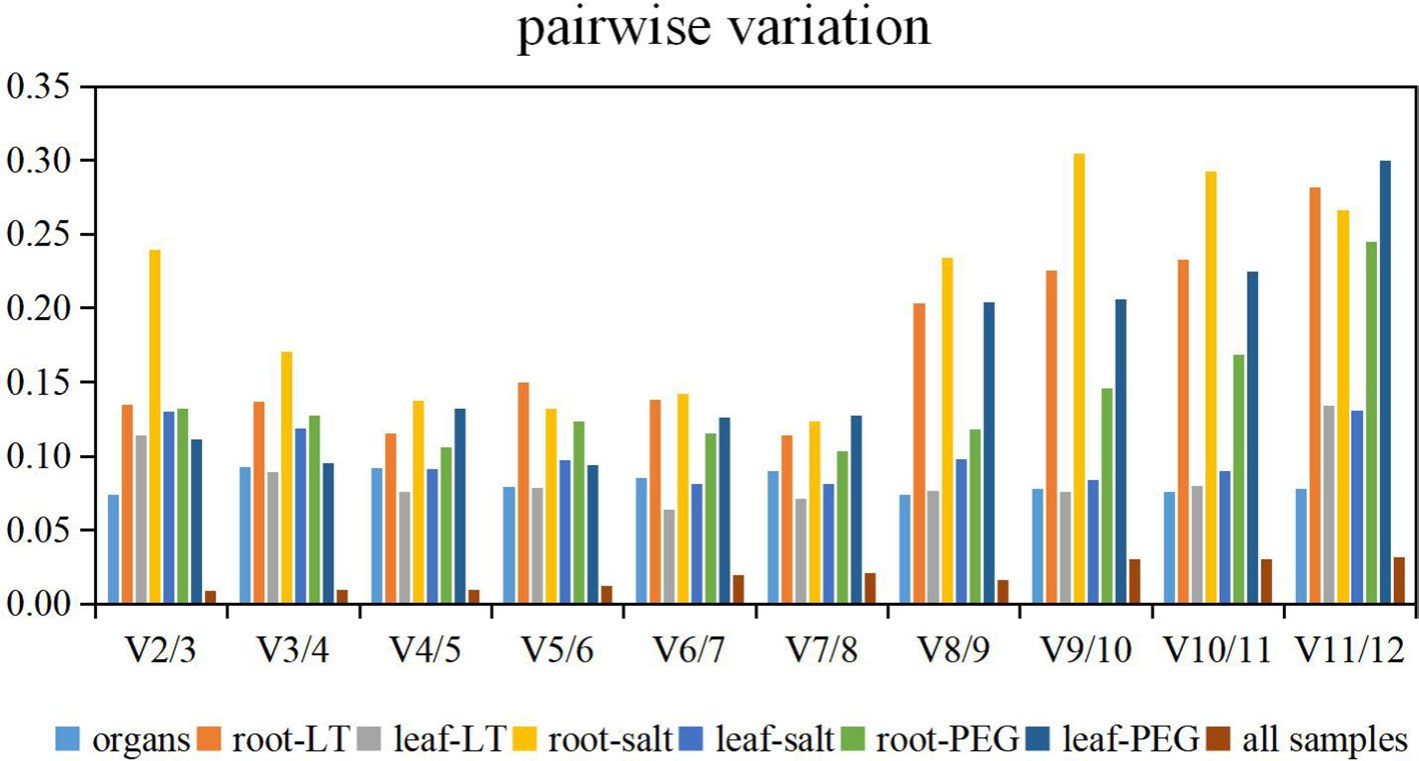
Pairwise V values of 12 candidate reference genes from Caucasian clover, as calculated by geNorm. The different treatments are marked with square frames of different colors. LT indicates the low-temperature treatment (4 °C), and PEG indicates the 10% PEG-6000 treatment; all samples of the four treatments were considered jointly as all samples, and organs were evaluated under normal conditions, as described below.

We also calculated the optimal number of reference genes according to the pairwise variation results (V_n/n+1_ values). The results showed that, with the exception of those of the roots of plants under salt stress (V_4/5_ = 0.13), all of the V_2/3_ pairwise variations calculated by geNorm were less than 0.15, and V_4/5_ was determined to be 0.13 for the roots of plants subjected to salt stress. These results suggested that the two most stable reference genes were adequate for qRT-PCR-based normalization across different experimental conditions and that an additional reference gene was not needed (Fig. 4).

### NormFinder analysis

In NormFinder analyses (Table 2), relatively low stability values indicate relatively stable reference genes. The results of the NormFinder analysis are listed in Table 2. The most stable reference genes differed among organs and stresses. The most stable reference genes included *RCD1* under salt stress, *PPIL3* under 10% PEG-6000 stress and *PTPMT1* under low-temperature stress across all the leaf samples. The most stable reference gene under salt and low-temperature stresses was *PP2A*, and that under 10% PEG-6000 stress was *PTPMT1* across all the root samples. The most stable reference genes across all the organs and samples were *EFTu-GTP* and *RBD*, respectively. The least stable reference genes across all the samples and conditions were *MAP* and *TMP*, respectively.

**Table 2:** Ranking of 12 candidate reference genes according to NormFinder.

Rank	Leaf-LT	Root-LT	Leaf-NaCl	Root-NaCl	Leaf-PEG	Root-PEG	Organs	All samples
1	PTPMT1	PP2A	RCD1	PP2A	PPIL3	PTPMT1	EFTu-GTP	MAP
2	BZIP	PPIL3	PPIL3	NLI2	PP2A	MAP	RBD	TMP
3	NLI1	EFTu-GTP	PTPMT1	PPIL3	NLI1	RBD	NLI2	EFTu-GTP
4	RCD1	RBD	PP2A	PTPMT1	RBD	PP2A	APA	NLI1
5	MAP	RCD1	RBD	EFTu-GTP	EFTu-GTP	BZIP	PPIL3	PTPMT1
6	RBD	APA	NLI1	RCD1	RCD1	PPIL3	PTPMT1	RCD1
7	EFTu-GTP	TMP	MAP	TMP	NLI2	APA	MAP	BZIP
8	PP2A	NLI2	NLI2	NLI1	MAP	NLI1	PP2A	PP2A
9	PPIL3	PTPMT1	TMP	APA	TMP	NLI2	NLI1	APA
10	APA	MAP	APA	RBD	PTPMT1	EFTu-GTP	BZIP	NLI2
11	TMP	BZIP	EFTu-GTP	MAP	APA	RCD1	RCD1	RBD
12	NLI2	NLI1	BZIP	BZIP	BZIP	TMP	TMP	PPIL3

### ∆Ct analysis

Relatively small ΔCt values of candidate reference genes indicate relatively high gene expression. As shown in Table 3, the ΔCt values differed among the stresses. In the organs, the smallest ΔCt values of the reference genes were 0.56, 0.56 and 0.58 for *EFTu-GTP*, *RBD* and *NLI2*, respectively, and the most unstable reference gene was *TMP*. However, the most stable reference genes were *EFTu-GTP* and *NLI1* across all the samples, and *PPIL3* was the most unstable reference gene overall. Among all the leaf samples, the most stable reference genes were *PTPMT1* under low-temperature stress and *PPIL3* under both salt stress and 10% PEG-6000 stress. Among all the root samples, the most stable reference genes were *EFTu-GTP* under low-temperature stress, *PP2A* under salt stress and *MAP* under 10% PEG-6000 stress.

**Table 3:** Stability of candidate reference genes according to the ΔCt method.

Item	RCD1	PPIL3	PP2A	NLI1	NLI2	EFTu-GTP	RBD	APA	PTPMT1	TMP	MAP	BZIP
Leaf-LT	0.68	0.84	0.81	0.68	1.68	0.81	0.72	0.88	0.63	1.04	0.73	0.66
Root-LT	1.20	1.18	1.14	3.47	1.25	1.13	1.18	1.24	1.29	1.25	1.33	2.61
Leaf-NaCl	0.71	0.71	0.80	0.85	0.93	1.20	0.82	1.01	0.77	1.02	0.87	1.65
Root-NaCl	1.68	1.61	1.49	1.86	1.51	1.69	3.40	2.70	1.63	1.82	3.45	3.50
Leaf-PEG	1.43	1.34	1.35	1.38	1.46	1.61	1.42	2.62	2.30	2.15	1.64	3.76
Root-PEG	2.11	1.15	1.16	1.28	1.50	1.75	1.13	1.22	1.17	3.05	1.10	1.14
Organs	1.00	0.69	0.88	0.88	0.58	0.56	0.56	0.61	0.71	1.03	0.77	0.88
All samples	0.17	0.38	0.20	0.16	0.23	0.16	0.31	0.27	0.17	0.17	0.17	0.18

### BestKeeper analysis

In BestKeeper analysis (Table 4), the ranking of candidate reference genes is evaluated according to CV and SD values, which are determined by Ct values. Relatively high stability of reference genes is represented by relatively low SD and CV values, and candidate reference genes are unsuitable for normalization and unstably expressed when the SD is > 1.

**Table 4:** Ranking of 12 candidate reference genes according to BestKeeper.

Rank	Leaf-LT			Leaf-NaCl			Leaf-PEG			Organs		
	Gene	CV	SD	Gene	CV	SD	Gene	CV	SD	Gene	CV	SD
1	EFTu-GTP	1.09	0.29	RCD1	1.07	0.26	PTPMT1	1.53	0.36	EFTu-GTP	0.31	0.08
2	RCD1	2.32	0.57	PP2A	1.11	0.25	EFTu-GTP	2.30	0.62	NLI2	0.44	0.11
3	NLI1	2.42	0.66	PPIL3	1.24	0.31	TMP	2.62	0.71	RBD	0.74	0.18
4	APA	2.48	0.61	PTPMT1	1.32	0.30	RCD1	2.82	0.70	APA	1.34	0.31
5	TMP	2.54	0.63	RBD	1.56	0.39	APA	3.16	0.83	MAP	1.46	0.32
6	BZIP	2.58	0.62	NLI2	1.99	0.53	PPIL3	3.27	0.85	NLI1	1.60	0.40
7	RBD	2.99	0.77	MAP	2.05	0.46	NLI2	3.47	0.99	PPIL3	1.96	0.47
8	PTPMT1	3.13	0.72	NLI1	2.13	0.56	PP2A	3.52	0.81	PTPMT1	2.02	0.44
9	PPIL3	3.18	0.81	APA	2.52	0.58	RBD	3.67	0.98	BZIP	2.21	0.50
10	MAP	3.21	0.88	TMP	3.02	0.73	MAP	3.79	0.90	PP2A	2.75	0.61
11	PP2A	3.73	0.87	BZIP	3.46	0.82	NLI1	6.30	1.74	RCD1	3.07	0.73
12	NLI2	4.53	0.98	EFTu-GTP	3.60	0.91	BZIP	9.95	2.35	TMP	3.08	0.73

Rank	Root-LT			Root-NaCl			Root-PEG			All samples		
	Gene	CV	SD	Gene	CV	SD	Gene	CV	SD	Gene	CV	SD
1	PP2A	1.04	0.23	NLI2	1.77	0.45	PPIL3	1.89	0.43	PTPMT1	0.02	0.01
2	RCD1	1.28	0.31	PP2A	1.87	0.41	PP2A	1.98	0.44	NLI1	0.03	0.01
3	PPIL3	1.40	0.35	RCD1	1.92	0.47	MAP	2.05	0.46	RCD1	0.09	0.02
4	TMP	1.81	0.46	EFTu-GTP	2.04	0.52	BZIP	2.10	0.47	BZIP	0.12	0.03
5	RBD	2.31	0.54	PPIL3	2.15	0.52	RBD	2.10	0.52	PP2A	0.19	0.04
6	APA	2.37	0.59	NLI1	3.02	0.80	APA	2.53	0.59	NLI2	0.27	0.07
7	PTPMT1	2.63	0.61	PTPMT1	3.07	0.67	PTPMT1	2.76	0.60	EFTu-GTP	0.28	0.07
8	NLI2	2.89	0.64	TMP	3.24	0.78	NLI2	2.78	0.71	TMP	0.43	0.11
9	MAP	2.92	0.76	APA	7.15	1.67	NLI1	2.92	0.81	MAP	0.48	0.11
10	EFTu-GTP	3.27	0.73	MAP	7.59	1.73	EFTu-GTP	3.20	0.80	APA	0.96	0.23
11	BZIP	6.88	1.62	RBD	7.86	1.99	RCD1	5.28	1.35	RBD	1.12	0.27
12	NLI1	7.95	2.27	BZIP	9.93	2.65	TMP	8.08	2.10	PPIL3	0.34	0.33

As shown in Table 4, among all the leaf samples, the most stable reference genes were *RCD1* under salt stress, *PTPMT1* under 10% PEG-6000 stress and *EFTu-GTP* under low-temperature stress. In the leaf samples of plants treated with 10% PEG-6000, the SD values of *NLI1* and *BZIP* were greater than 1, which indicated that the reference genes could not be used for normalization. Among all the root samples of the plants, the most stable reference genes were *NLI2* under salt stress, *PPIL3* under 10% PEG-6000 stress and *PP2A* under low-temperature stress. Among the different organs, the most stable reference gene was *EFTu-GTP*, and *PTPMT1* was the most stable reference gene across all the samples. Candidate reference genes with an SD > 1 were identified in the roots of plants treated with salt, 10% PEG-6000 and low temperature; these genes could not be used for normalization.

### RefFinder analysis

The online tool RefFinder was used to determine the comprehensive ranking of the candidate genes analyzed by the other four methods (geNorm, NormFinder, BestKeeper and the ΔCt method). The results of RefFinder also showed that the comprehensive rankings of the reference genes differed under the various stresses and among the different organs (Supplementary Table S2 and Table 5). The most stable reference genes were *RCD1*, *NLI1* and *APA* in most of the samples under various stresses and in the different organs. In contrast, *EFTu-GTP*, *BZIP*, *NLI2*, *PP2A* and *APA* were the most unstable reference genes under various stresses and in different organs. Compared with the most unstable reference genes, the most stable reference genes were more consistent. We used RefFinder to calculate the geometric mean of the stability rankings obtained from the analysis of geNorm, NormFinder, BestKeeper and the ∆Ct method and obtained a comprehensive ranking (Supplementary Fig. S3).

**Table 5:** Most stable and least stable reference genes according to RefFinder.

Leaf-LT		Root-LT		Leaf-NaCl		Root-NaCl		Leaf-PEG		Root-PEG		Organs		All samples	
Most	Least	Most	Least	Most	Least	Most	Least	Most	Least	Most	Least	Most	Least	Most	Least
APA	PP2A	RCD1	EFTu-GTP	RCD1	APA	NLI1	NLI2	NLI1	BZIP	RCD1	BZIP	RCD1	EFTu-GTP	RCD1	EFTu-GTP
EFTu-GTP		NLI2		PPIL3		RCD1		NLI2		PPIL3		NLI2		NLI2	

### Reference gene validation

To validate the ranking of the 12 candidate reference genes of Caucasian clover, the expression levels of the *WRKY* gene in the roots of plants under low-temperature treatment (Supplementary Table S3) and in the roots of plants under salt treatment (Supplementary Table S4) were normalized. WRKY proteins compose a large family of transcription factors involved in the abiotic stress response^31^, and many *WRKY* genes have been identified in *Oryza sativa*^32^, *Hordeum vulgare*^33^, *Cucumis sativus*^34^ and citrus species^35^.

On the basis of the comprehensive analysis of the results from geNorm, NormFinder, BestKeeper, and the ΔCt method by RefFinder, the two most stable reference genes and the least stable reference genes under different treatment conditions were selected. In root samples from plants subjected to salt and low-temperature treatments, the expression of *WRKY* at 0 h was assumed to be 1 and was used to compare the relative expression of genes from the samples at other time points.

**Figure 5:**
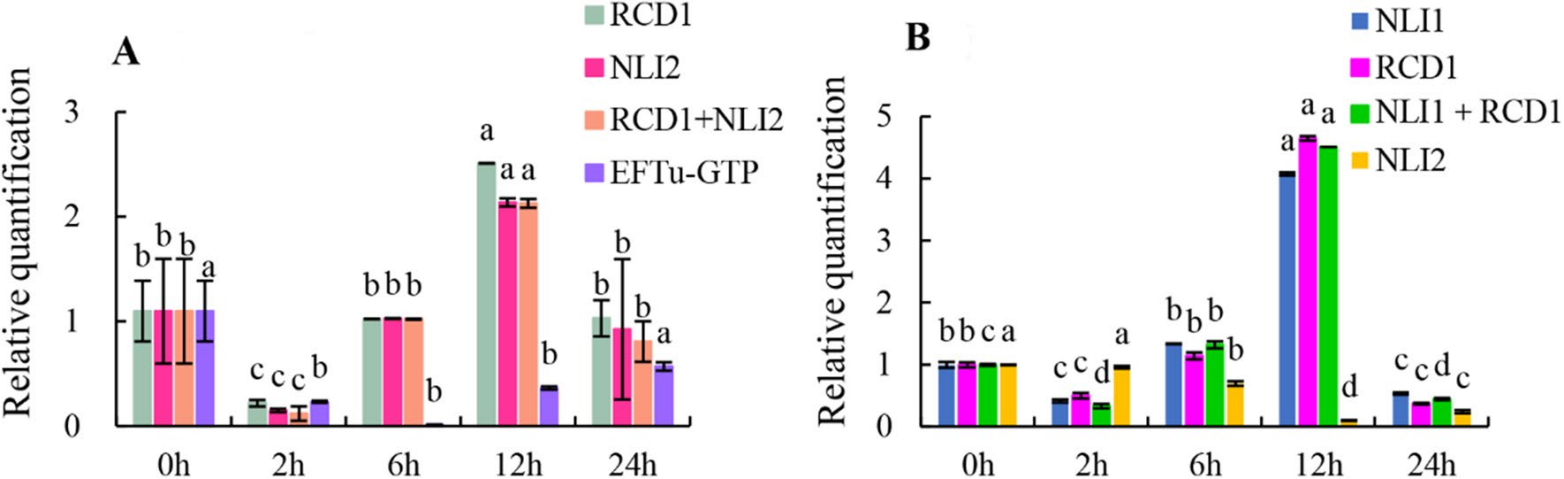
The relative expression level of *WRKY* was determined by the use of select reference genes, including the most or least stable reference genes, for normalization in the roots. (**A**) Relative quantification of *WRKY* expression in the roots of plants under low-temperature stress. (**B**) Relative quantification of *WRKY* expression in the roots of plants under salt stress. All the materials were treated for 0, 2, 6, 12 or 24 h. The error bars represent the standard errors of the means of three biological replicates. Statistical analysis was performed at different times for the same reference gene. The means with different letters are significantly different from one another (P < 0.01).

As shown in Fig. 5, in the roots of plants under low-temperature stress, the two most stable reference genes, *RCD1* and *NLI2* (including *RCD1* + *NLI2*), and the least stable reference gene, *EFTu-GTP*, according to the results of the comprehensive evaluation were used to calibrate the relative expression of the *WRKY*, *RCD1*, and *NLI2* target genes, respectively. In addition to the expression pattern of the *RCD1* + *NLI2* combination, which was used as a reference gene, the expression pattern of *WRKY* was consistent. When the least stable reference gene (*EFTu-GTP*) was used to correct the target gene, the expression pattern greatly differed from the above expression pattern. In the roots of plants under salt stress, the relative expression of the *WRKY* target gene was validated using the two best reference genes, *NLI1* and *RCD1*; the *NLI1* + *RCD1* combination; and the least stable reference gene, *NLI2*. When *NLI1*, *RCD1*, and the *NLI1* + *RCD1* combination were used as references, the expression pattern of WRKY was consistent. When the least stable reference gene (*NLI2*) was used to validate the target gene, the expression pattern was very different from the above expression pattern.

In the roots of plants under low-temperature treatment, the least stable reference gene was *EFTu-GTP*, and the most stable reference genes were *RCD1* and *NLI2*. In the roots of plants under salt stress, the least stable reference gene was *NLI2*, and the most stable reference genes were *NLI1* and *RCD1*. We also used the combination of the most stable reference genes for validation.

## Discussion

As an advanced, accurate and commonly used research tool, qRT-PCR is pivotal for quantifying the relative expression levels of target genes^36^. qRT-PCR has been used to quantify relative levels of gene expression on the basis of normalization to the expression of stable reference genes^37^. Unstable reference genes can substantially affect the results of such analyses and can even lead to erroneous conclusions^38^. Primer amplification efficiency represents the amplicon doubling rate during the PCR process, which also affects the accuracy of qRT-PCR^16^. A good primer has an E value ranging from 90 to 105%^23^. In this study, the E values of all the primers used were in this range, ensuring the accuracy of the qRT-PCR data in this study.

After consulting previous studies of other species and searching our transcriptomic database, we selected 12 candidate reference genes for evaluation. No reference genes suitable for different tissues or organs of Caucasian clover related to growth and development and stress conditions were identified. In general, the most stable genes in all the samples were *RCD1*, *PPIL3*, *NLI1* and *NLI2*, whereas *bZIP* and *EFTu-GTP* were the most unstable reference genes. *bZIP* and EFTu-GTP encode a transcription factor and an elongation factor Tu GTP-binding domain-containing protein, respectively. It is very easy to draw erroneous conclusions due to random effects caused by heterogeneity of the samples. Therefore, it is not recommended to use *bZIP* or *EFTu-GTP* as a reference gene in such experiments. *RCD1* is needed to maintain cells in a division-competent state and to regulate division plane placement^39^. Reports on *PPIL3*, *NLI1* and *NLI2* in plants are scarce. *PP2A* was evaluated in four strains of *Auricularia cornea*^40^ at different developmental stages, and its comprehensive ranking was 3. The rankings generated from the four algorithms were incomplete, and the results were confirmed in *Rhododendron*^20^, *Taihangia rupestris*^41^ and *Baphicacanthus cusia*^42^. In the leaves and fruits of *Lagenaria siceraria*^43^, the reference gene *CYP* was the most unstable according to geNorm.

Nearly all of the relevant studies have revealed that the use of more than one reference gene for normalization yields more accurate results^44,45^. In most cases, the gene stability results calculated by geNorm and NormFinder were similar (Fig. 3 and Table 2). The best number of reference genes used for normalization was calculated by geNorm, and the results showed that, with the exception of the value from root samples of plants under salt stress, all the V_2/3_ values were below the threshold (0.15). A V_2/3_ value of < 0.15 indicated that the optimal number of reference genes for normalization was two, and a V_4/5_ value of < 0.15 in the roots of plants under salt stress indicated that the best number of reference genes for normalization was four, which was not ideal. However, the M values calculated by geNorm can also be used to evaluate the stability of reference genes; the results showed that only three of the studied reference genes were unstable, while the M values of the other reference genes were below 1.50. Thus, V scores cannot be used as the only index. On the basis of the results of this study, we suggested that using the most stable reference gene for normalization may be a better choice for the roots of plants under salt stress conditions. However, the gold standard of qRT-PCR is the use of at least four reference genes to determine the deviation of a single reference gene^38^. Using four algorithms (geNorm, NormFinder, BestKeeper and the ΔCt method), we found that the rankings of all the reference genes differed among the results of the different algorithms. This variation was expected, however, because the four approaches involve the use of different calculation algorithms, each of which has been verified in many previous studies^39–41^.

In this study, for further verification of the best reference genes, we compared the CVs of the FPKMs of 12 candidate reference genes between the control and different organs in our RNA sequencing (RNA-seq) data (Fig. S2). At the same time, the CVs of the 12 candidates were also compared with our results obtained by geNorm, NormFinder and BestKeeper (Fig. S2), and the overall results were generally consistent. However, this did not undermine the final results of the experiments, because the sequencing data were used only for verification in subsequent validation experiments instead of being directly used to draw conclusions. For this reason, the data could be considered only preliminary, which indicated the trends the FPKM CV values of 12 candidate reference genes. We validated the selected reference genes according to the relative expression of *WRKY* in the roots of plants under low-temperature stress and that of *WRKY* in the roots of plants under salt stress. The results shown in Fig. 3 confirm that the candidate reference genes were applicable and stably expressed in Caucasian clover. The results also indicated that the most stable reference genes differed among organs and among treatments, even within the same organ.

## Conclusion

Twelve candidate reference genes were selected for qRT-PCR standardization evaluation, and five different statistical methods were used. The results showed that *RCD1*, *PPIL3*, *NLI1* and *NLI2* were the most stable reference genes, while *bZIP* and *EFTu-GTP* were the most unstable reference genes. The stable reference genes identified in this report will enhance the accuracy of qRT-qPCR-based analysis of target gene expression and can be used to study related functional genes in Caucasian clover.

## Supplementary Information

Supplementary Figures.

Supplementary Tables.
