# Supplementary material for: Comprehensive evaluation of candidate reference genes for quantitative real-time PCR-based analysis in Caucasian clover
Source: Sci Rep. 2021 Feb 8;11:3269. doi: 10.1038/s41598-021-82633-2 (PMC7870939; doi:10.1038/s41598-021-82633-2)
Supplement: Supplementary file 1 — Supplementary Figures. [file 41598_2021_82633_MOESM1_ESM.docx]

Comprehensive evaluation of candidate reference genes for quantitative real-time PCR-based analysis in caucasian clover

Author list:

Xiujie Yin1, Taotao He2, Kun Yi3, Yihang Zhao4, Yao Hu5, Jiaxue Liu6, Xiaomeng Zhang7, Lingdong Meng8, Lina Wang9, Haoyue Liu10, Yonggang Li11, Guowen Cui*

1College of Animal Science and Technology, Northeast Agricultural University, No. 600 Changjiang Street, Xiangfang District, Harbin City, Heilongjiang, China;

2College of Animal Science and Technology, Northeast Agricultural University, No. 600 Changjiang Street, Xiangfang District, Harbin City, Heilongjiang, China;

3College of Animal Science and Technology, Northeast Agricultural University, No. 600 Changjiang Street, Xiangfang District, Harbin City, Heilongjiang, China;

4College of Animal Science and Technology, Northeast Agricultural University, No. 600 Changjiang Street, Xiangfang District, Harbin City, Heilongjiang, China;

5College of Animal Science and Technology, Northeast Agricultural University, No. 600 Changjiang Street, Xiangfang District, Harbin City, Heilongjiang, China;

6College of Animal Science and Technology, Northeast Agricultural University, No. 600 Changjiang Street, Xiangfang District, Harbin City, Heilongjiang, China;

7College of Animal Science and Technology, Northeast Agricultural University, No. 600 Changjiang Street, Xiangfang District, Harbin City, Heilongjiang, China;

8College of Animal Science and Technology, Northeast Agricultural University, No. 600 Changjiang Street, Xiangfang District, Harbin City, Heilongjiang, China;

9College of Animal Science and Technology, Northeast Agricultural University, No. 600 Changjiang Street, Xiangfang District, Harbin City, Heilongjiang, China;

10College of Animal Science and Technology, Northeast Agricultural University, No. 600 Changjiang Street, Xiangfang District, Harbin City, Heilongjiang, China;

11College of Animal Science and Technology, Northeast Agricultural University, No. 600 Changjiang Street, Xiangfang District, Harbin City, Heilongjiang, China;

*College of Animal Science and Technology, Northeast Agricultural University, No. 600 Changjiang Street, Xiangfang District, Harbin City, Heilongjiang, China;

* Corresponding author mail

cuigw603@126.com (Guowen Cui)

**Figure legends**

S1 Fig. Specificity of Caucasian clover reference gene primer pairs used for RT-qPCR amplification. Figs. 1, 2, 3, 4, 5 and 6 indicate the reference genes of *APA*, *PTPMT1*, *TMP*, *MAP*, *BZIP*, and *WRKY*, respectively.

S2 Fig. Stability rankings of candidate genes according to FPKM CVs from RNA-seq.

S3 Fig. Comprehensive rankings of geNorm, NormFinder, BestKeeper and ∆Ct from RefFinder analysis.

S1 Fig.





S2 Fig.

S3 Fig.
